# Impact of body composition analysis on male sexual function: A metabolic age study

**DOI:** 10.3389/fendo.2022.1050441

**Published:** 2023-01-04

**Authors:** Ahmad Majzoub, Haitham Elbardisi, Sarah Madani, Kristian Leisegang, Mohamed Mahdi, Ashok Agarwal, Ralf Henkel, Kareim Khalafalla, Sami ElSaid, Mohamed Arafa

**Affiliations:** ^1^ Department of Urology, Hamad Medical Corporation, Doha, Qatar; ^2^ Clinical Urology, Weill Cornell Medicine -Qatar, Doha, Qatar; ^3^ Department of Biology and Physiology or Organisms, University of Science and Technology Houari Boumediene, Algiers, Algeria; ^4^ School of Natural Medicine, Faculty of Community and Health Sciences, University of the Western Cape, Bellville, South Africa; ^5^ Case Western Reserve University, Moreland Hills, OH, United States; ^6^ Department of Medical Bioscience, University of the Western Cape, Bellville, South Africa; ^7^ Department of Metabolism, Digestion and Reproduction at Imperial College London, London, United Kingdom; ^8^ LogixX Pharma, Theale, United Kingdom; ^9^ Andrology Department, Cairo University, Cairo, Egypt

**Keywords:** body mass index, basal metabolic rate, erectile dysfunction, hypogonadism, metabolic age

## Abstract

**Introduction:**

Metabolic Age (MetAge) and body composition analysis may reflect an individual’s metabolic status, which is believed to influence male sexual and gonadal functions. Although erectile dysfunction (ED) and hypogonadism are increasingly prevalent with age, they are also detected among younger men. This study aims to assess the impact of MetAge and body composition on male sexual and gonadal status overall, and particularly in men younger than 40 years of age.

**Methods:**

This was a cross-sectional study of 90 male healthcare workers, between the ages of 18-55, randomly selected based on their corporation numbers. In addition to Bioelectric Impedance Analysis, subjects were requested to fill the International Index of Erectile Function questionnaire (IIEF-5) and to provide an early morning serum testosterone (T) sample.

**Results:**

The mean participants’ age was 39.4 ± 9.4 years, MetAge was 45.54 ± 10.35 years, serum T level was 13.68 ± 4.49 nmol/L and BMI was 28.8 ± 4.7 kg/m^2^. Significant negative correlations were obtained between serum T, MetAge, body weight and fat composition. Significant negative correlations between the IIEF-5 score, MetAge, and fat composition, were only reported in subjects <40 years of age. Significantly lower T levels (p=0.002), significantly older MetAge (p=0.034), and higher BMI (p=0.044) and degree of obesity (p=0.042) were observed in participants <40 years with erectile dysfunction (ED) compared to their counterparts without ED.

**Discussion:**

MetAge and body composition parameters significantly impact the androgenic state. ED in men <40 years is associated with lower T levels, older MetAge and higher BMI and degree of obesity.

## Introduction

The human body is composed of balanced percentages of fat, muscle, bone, water and connective tissue (1). This balance is a potential indicator of the state of health of each person, in relation to his or her origin, chronological age, sex, diet, physical activities as well as their lifestyle (2). These variables are known to influence body weight and body fat composition and consequently body mass index (BMI) and basal metabolic rate (BMR) (1, 2).

An individual’s BMR, defined by the number of calories burned during the awake resting state determines the metabolic age (MetAge). The latter is obtained by comparing the BMR of an individual to the BMR average of his/her chronologic age group in the general population (3). Body fat is essential for maintaining optimal body temperature, as well as protecting joints and internal organs (4, 5). However, it is well known that an increase in fat mass, more precisely in the visceral region (6), is associated with a disturbance of internal homeostasis, endocrine and metabolic imbalance and a cascade of inflammations causing functional impairments of different organs and systems (7). Indeed, many diseases are diagnosed in overweight and obese subjects such as cardiovascular disease, type-2 diabetes, dyslipidemia, metabolic syndrome, as well as other pathologies whose complications can result in different types of cancer or even mortality in some cases (8). Body composition and fat mass can also interfere with reproductive function (9). Metabolic disturbances caused by excess weight such as metabolic syndrome, increased adipocyte secretions, hyperinsulinemia and dysregulation of steroid metabolism, have repercussions at central and peripheral levels leading to reproductive disorders of varying intensity (10, 11). Hypogonadism and erectile dysfunction (ED), defined by the inability to obtain and maintain an erection allowing satisfactory sexual intercourse (12), are examples of reproductive conditions that have been strongly correlated with obesity (13). While these sexual disorders are generally more prevalent among aging men (14), several studies have shown them to be not uncommon in younger generations holding poor metabolic status and obesity as the responsible risk factors for such an observation (15, 16).

Evaluation of the body composition is integral to weight reduction programs and might help in understanding the risks to sexual disorders that are linked to excessive fat deposition and weight gain. Several methods have been utilized for body composition analysis including dual-energy X-ray absorptiometry, waist circumference, visceral adiposity index, anthropometric measurements and bioelectric impedance analysis (17, 18). These methods provide a detailed understanding of the body composition ratios and hence are appealing means that can be used to investigate disorders associated with impaired metabolism (17, 18). While several studies investigated the impact of obesity on reproductive function, very few have assessed the relationship between individual body composition parameters and male sexual and gonadal health. Therefore, the objectives of this study were to (1) investigate the overall relationship between body composition parameters, serum testosterone levels and erectile function; and (2) compare testosterone levels, MetAge and body composition analysis results in men younger than 40 of age with and without ED.

## Material and methods

### Study design, participants and setting

This cross-sectional study was conducted at tertiary medical center between March, 2016 and March, 2017. A total of 120 male healthcare providers, randomly selected based on their corporation numbers through computer-generated randomization tables, were electronically invited to participate in this study.

The Institutional Review Board approved the study protocol and informed consents were signed by participants before enrollment in the study.

Adult, sexually active men >18 years of age were included in this study. The exclusion criteria were: subjects [1] receiving treatment with phosphodiesterase inhibitors or any other erectogenic supplements; [2] taking testosterone replacement therapy or other medications to increase endogenous testosterone levels including estrogen receptor modulators, aromatase inhibitors and gonadotrophins; [3] known to have endocrine abnormalities that may impact sexual function including hyperprolactinemia, pan-hypopituitarism and hypothyroidism; [4] with debilitating medical diseases including end-stage renal disease, liver failure and congestive heart failure; [5] who were receiving recreational drugs or alcoholic; [6] who had a history of infertility due to a male factor including cryptorchidism, orchitis, and testicular torsion or trauma.

### Study procedures

All participants reported in the morning following an overnight fast of 12 hours and abstinence from for at least 24 hours, to provide an early morning serum testosterone sample, and to undergo Bioelectric Impedance Analysis according to the protocol validated by Lukaski et al., 1986 to assess body composition (19). Serum testosterone levels (reference range 10.4 – 34 nmol/L) were analyzed using the immunoassay chemiluminescence method, Architect i1000SR^®^ (Abbott systems, Illinois, USA). Hypogonadism is defined in subjects who had a serum testosterone level <10.4nmol/L. Subjects were also requested to fill in the English five-item version of the International Index of Erectile Function questionnaire-5 (IIEF-5) a brief, reliable, and valid self-administered questionnaire containing five questions that have been widely used in many countries to detect the presence and severity of ED (score range: 5-25, severe ED: 5-7, moderate ED: 8-11, mild-moderate ED: 12-16, mild ED: 17-21, no ED: 22-25) (20). All participants had a higher level of education and good command of the English language.

The metabolic status of participants was assessed using the TANITA body analyzer (TBF-410GS) (Arlington Heights, Illinois, USA) which is a reliable method for assessing body composition (21). Measurements were performed with subjects fasting for a minimum of 8 hours, wearing light clothing and no shoes or socks. Quality control for all measurements was monitored regularly. Results of the body composition analysis include weight, body fat %, body mass index (BMI), degree of obesity, MetAge and visceral fat. The degree of obesity is an estimate of overweight in relation to the norm in percentage and is calculated using the following formula: [(weight – ideal weight)/ideal weight] x 100.

### Data, variables

Risk factors for sexual dysfunction such as diabetes mellitus, hypertension, coronary artery disease, dyslipidemia and smoking were assessed through interview.

Demographic data (age), clinical (testosterone, results of IIEF-5) and body composition results (noted above) were also collected. Participants were divided according to their chronologic age into two groups: (i) group 1 with participants whose age was under 40 years (18-39), and (ii) group 2 with participants whose age was equal or above 40 years (40-65) (22–24).

### Statistical analysis

The distribution of the data was assessed using the Shapiro-Wilk test. All numerical data were presented as means ± Standard Deviation (SD), while categorical data were presented with frequencies (%).

The relationship between participants’ sexual and androgenic state and their body composition parameters were assessed using Pearson’s correlations. Student T-test was used to compare testosterone levels and results of body composition analysis between men <40 years of age with or without sexual dysfunction. Clustered box plots were used to picture differences in chronologic and MetAge in men with serum testosterone (<10.4 or ≥ 10.4) and with or without ED. A p-value <0.05 was considered statistically significant. Statistical analysis of collected data was performed using SPSS version 25 (IBM, Armonk, NY, USA).

## Results

Among the 120 participants, only 90 subjects met the inclusion and the exclusion criteria for this study. None of the participants had undergone any bariatric surgery. The average chronologic age of the population was 39.41 ± 9.39 years [30-68 years], of which 53 individuals were under the age of 40 and 37 individuals were over the age of 40. A low testosterone level was detected in 18 subjects (20%) and risk factors were detected in 41 (45.6%) subjects. Risk factors were more prevalent among men ≥40 compared with men <40 years of age (54.1% vs. 39.6%). The characteristics of the study population including demographic, clinical and body analysis data are shown in **Table 1**.

**Table 1:** Characteristics of the study population: whole population, participants < and ≥ 40 years of age.

Demographic and clinical data	Whole population (n=90)	Participants <40 years of age (n=53)	Participants ≥40 years of age (n=37)
Age (years)	39.41 ± 9.39	32.77 ± 2.74	48.92 ± 7.02
Testosterone (nmol/L)	13.68 ± 4.49	12.87 ± 4.45	14.84 ± 4.34
IIEF-5 score	20.91 ± 2.86	22.38 ± 2.34	20.43 ± 3.12
Mild -Moderate ED	7 (7.7)	3 (5.6)	4 (10.8)
Mild ED	41 (45.5)	21 (39.6)	20 (54.1)
No ED	42 (46.6)	29 (75.5)	13 (35.1)
Risk Factors	41 (45.6)	21 (39.6)	20 (54.1)
Diabetes Mellitus	7 (7.8)	1 (1.9)	6 (16.2)
Hypertension	12 (13.3)	4 (7.5)	8 (21.6)
Smoking	29 (32.2)	17 (32.1)	12 (32.4)
Coronary artery disease	1 (1.1)	0 (0)	1 (2.7)
Body composition analysis			
Metabolic Age (Years)	45.54 ± 10.35	42.04 ± 8.14	50.57 ± 11.67
Weight (kg)	88.28 ± 17.8	89.49 ± 16.87	86.54 ± 19.14
BMI (kg/m^2^)	28.8 ± 4.77	29.02 ± 4.49	28.49 ± 5.18
Fat (%)	26.03 ± 6.82	25.87 ± 5.84	26.27 ± 8.09
Fat mass (kg)	23.74 ± 9.88	24.27 ± 9.97	22.97 ± 9.8
Visceral fat (rate)	10.52 ± 4.1	9.57 ±3.91	11.89 ± 4.01
Degree of obesity (%)	31.12 ± 21.51	32.06 ± 20.33	29.78 ± 23.31
Muscle mass (kg)	61.42 ± 10.05	62.53 ± 8.45	59.84 ± 11.92
Bone mass (kg)	61.42 ± 10.05	3.22 ± 0.51	3.19 ± 0.61
BMR (Kj)	8050.77 ± 1280.03	8199.34 ± 1203.01	7837.95 ± 1371.54

IIEF-5, International Index of Erectile Function-5 item version; ED, Erectile Dysfunction; BMI, Body Mass Index; BMR, Basal Metabolic Rate.

ED was reported by 48 (53%) subjects, with an average IIEF-5 score of 20.04 ± 3.0.

**Table 2** reports the correlations between the different continuous study variables.

**Table 2:** Pearson correlation between variables in the whole study population and the subjects < or ≥ 40 years old.

r	Testosterone			IIEF score		
	Whole population (n=90)	<40 years (n=53)	≥ 40 years (n=37)	Whole population (n=90)	<40 years (n=53)	≥ 40 years (n=37)
Testosterone (nmol/L)	1	1	1	0.135	-0.296*	0.049
Age (years)	0.186	-0.145	0.086	-0.203	-0.057	-0.084
Metabolic age (years)	-0.267*	-0.347*	-0.467**	-0.090	-0.304*	0.253
IIEF score	0.135	-0.296*	0.049	1	1	1
Weight (Kg)	-0.391**	-0.372**	-0.402*	0.034	-0.190	0.245
BMI (Kg/m^2^)	-0.430**	-0.389**	-0.483**	-0.065	-0.283*	0.142
Fat %	-0.474**	-0.472**	-0.530**	-0.080	-0.259	0.087
Fat mass (Kg)	-0.477**	-0.447**	-0.518**	-0.070	-0.291*	0.174
Visceral fat (rate)	-0.377**	-0.441**	-0.505**	-0.151	-0.276*	0.106
Degree of obesity (%)	-0.439**	-0.394**	-0.499**	-0.067	-0.288*	0.145
Muscle mass (Kg)	-0.192	-0.210	-0.132	0.108	-0.115	0.259
Bone mass (Kg)	-0.178	-0.033	-0.320	0.098	-0.083	0.239
BMR (Kj)	-0.296**	-0.237	-0.326*	0.082	-0.136	0.265

■: Negative correlation, ■: Positive correlation, (*): Significant correlation P< 0.05, (**): highly significant correlation P<0.001, IIEF, International Index of Erectile Function; BMI, Body Mass Index; BMR, Basal Metabolic Rate; r, Pearson correlation coefficient.

Overall, a significant negative correlation was detected between testosterone and metabolic age, weight and BMI as well as various fat body composition elements (Fat%, fat mass, visceral fat and degree of obesity). On subgroup analysis these correlations were stronger in men ≥40 years of age.

With regards to the IIEF-5 score, no significant correlations were observed with other variables among participants, overall. However, significant negative correlations were observed with testosterone, MetAge, BMI, fat mass, visceral fat and degree of obesity only in men < 40 years of age.

Comparison of demographic, clinical and body composition analysis data in subjects < 40 years of age with/without ED was established (**Table 3**). ED was reported by 24 subjects who were < 40 years old, while the remaining 29 subjects had no ED with the IIEF-5 score.

Participants younger than 40 with ED had significantly lower T levels (10.88 ± 4.05 vs. 14.52 ± 4.14, p=0.002) compared to their counterparts without ED. Older MetAge was observed in ED vs no ED participants as well (44.63 ± 6.9 vs 39.9 ± 8.59, p=0.034) (**Table 3**).

**Table 3:** Comparison of demographic, clinical and body composition analysis data in subjects < 40 years of age with/without erectile dysfunction.

	No ED (n=29)	ED (n=24)	P value
Testosterone (nmol/L)	14.52 ± 4.14	10.88 ± 4.05	<0.01*
Weight (Kg)	86.42 ± 15.56	93.2 ± 17.97	0.14
Fat (%)	24.52 ± 5.95	27.5 ± 5.38	0.06
Fat mass (Kg)	21.88 ± 8.85	27.17 ± 10.66	0.07
Muscle mass (Kg)	61.41 ± 8.12	63.88 ± 8.82	0.29
Bone mass (Kg)	3.21 ± 0.41	3.29 ± 0.46	0.48
BMR (Kj)	8022.03 ± 1128.98	8413.58 ± 1277.82	0.24
Metabolic age (Years)	39.9 ± 8.59	44.63 ± 6.9	0.03*
Visceral fat (rate)	8.69 ± 3.82	10.63 ± 3.83	0.07
BMI (Kg/m^2^)	27.9 ± 4.42	30.38 ± 4.28	0.04*
Degree of obesity (%)	26.93 ± 20.12	38.25 ± 19.2	0.04*
Risk factors, n (%)	14 (48.27)	7 (29.17)	0.13

Independent t- test and Chi squared test, *: significant result p<0.05.

BMI, body mass index; BMR, basal metabolic rate.

**Figure 1:**
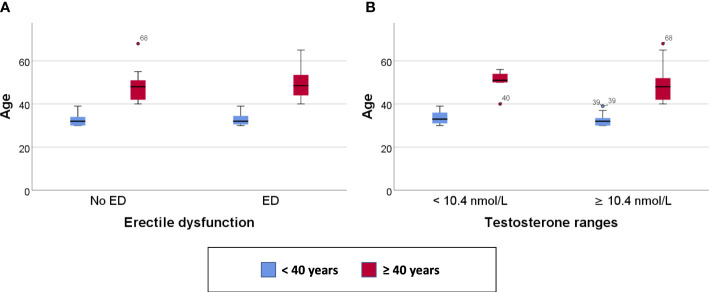
Clustered Box plot for: Age by sexual function **(A)** and serum testosterone level **(B)** in participants < or ≥ 40 years of age.

**Figure 2:**
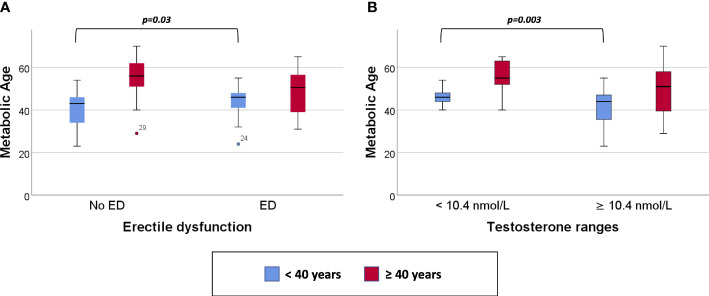
Clustered Box plot for: Metabolic Age by sexual function **(A)** and serum testosterone level **(B)** in participants < or ≥ 40 years of age.

Clustered box plots for chronologic age (**Figure 1**) and MetAge (**Figure 2**) by sexual function and testosterone levels stratified according to the study groups were drawn.

In subjects < 40 years of age, the mean MetAge was significantly higher in those with hypogonadism or with ED compared to their counterparts with normal testosterone levels or normal sexual function (**Figure 2**). No significant differences were noted for MetAge in men ≥40 years of age. Furthermore, no significant differences were noted in the chronologic age of men with low/normal testosterone or with/without ED among the two study groups (**Figure 1**).

## Discussion

This study revealed that body composition parameters including MetAge can significantly influence the sexual and gonadal health of men. More importantly, the increase in body mass index and MetAge may be associated with sexual dysfunction in men younger than 40 years of age.

Significant negative correlations were obtained between serum testosterone level and metabolic age, body weight and degree of fat deposition. Many studies have confirmed the presence of a close association between testosterone deficiency and obesity (25). The excessive secretion of leptin and pro-inflammatory cytokines by adipose tissue as well as the prevailing state of insulin resistance in obese men exert a negative impact on the hypothalamic-pituitary-gonadal axis ultimately reducing the secretion of gonadotropins (26). At the peripheral level, the increased production of leptin may further reduce the receptivity of Leydig cells to LH (27). While insulin resistance decreases hepatic sex hormone-binding globulin production and increases the availability of free testosterone (26, 28, 29), it thereby renders free testosterone a substrate for excessive aromatization to estradiol in adipose tissues (26). Estradiol consequently exhibits a negative effect on the hypothalamic-pituitary function and further aggravates the process (26). The European Male Aging Study showed that a decline in testosterone was observed in 73% of overweight or obese men; the serum testosterone in men with a BMI> 30 kg/m^2^ was on average 5 nmol/l lower to those of normal weight (30). In a cross-sectional study of young non-diabetic obese men, hypoandrogenemia was directly associated with adiposity (31).

Our results showed that in men < 40 years of age, the IIEF-5 score was significantly negatively correlated with serum testosterone levels, MetAge, BMI and degree of fat deposition. On the other hand, this finding was not obtained in men ≥ 40 years of age. This may be explained by the higher incidence of risk factors among men ≥ 40 years old, suggesting that the presence of risk factors in this population may undermine the effect of body composition parameters on sexual function which was observed in the younger age group. Age has been confirmed to be an independent risk factor for ED (32). Older men, are prone to systemic diseases such as hypertension, cardiovascular disease, type-2 diabetes or even psychoneurological diseases and are hence more likely to develop ED (33). However, some studies have suggested that ED in young men may be more frequent than we thought and could be attributed to life style exposures including smoking, alcohol or drug intake (34) as well as alterations in metabolic profiles of these individuals. Several studies have explored the relationship between various body composition parameters and sexual dysfunction in men. Visceral fat, represented by the visceral adiposity index which is a mathematical parameter obtained using waist circumference, BMI, serum triglyceride and high-density lipoprotein levels, was found to be significantly higher in men with ED in comparison to those without ED (35). Body fat mass, on the other hand, had a U-shaped relationship with erectile function in a study of Korean men indicating that worse sexual performance was reported by men with either too little or too much fat mass (36). A recent observational study by Molina-Vega et al. (2020) established an association between ED and obesity in a group of young non-diabetic obese men between the ages of 18-49 years (37). The authors revealed that the severity of ED was directly related to an increase in BMI, metabolic syndrome components, fat mass and lipid balance. While these results do corroborate with our findings, their study may have a selection bias as it only included obese patients who may be predisposed to other risk factors of ED and did not include a non-obese control group to confirm the association. In our, randomly selected study participants, we compared serum testosterone levels and body composition parameters between 2 equally sized groups <40 years of age with/without ED. We reported significantly lower serum testosterone, older MetAge and higher BMI and degree of obesity in men with ED compared to those without ED.

The impact of hypogonadism on the vascular tone has been investigated and reports have established a link between endothelial dysfunction and testosterone deficiency predisposing patients for ED (38). Hypogonadism may manifest with decreased production of nitric oxide (NO) synthetase thereby reducing the NO levels in the vascular endothelium (10, 39). This in addition to an upregulation in vasoconstrictor levels including endothelin-1 and pro-inflammatory factors (IL-6, CRP) result in impairment of cavernous smooth muscle hemodynamic properties leading to altered relaxation, or in other words, ED (11, 40).

This study was not without limitations. The study participants were healthcare workers who are not necessarily representative of the general population. Furthermore, ED was subjectively assessed by the participants who might under- or overestimate their sexual performance. HBA1c was not assessed and therefore it is unknown whether some of the participants might be pre-diabetic or with insulin resistance. The body composition analysis results were as valid as the accuracy of the machine utilized. Despite the fact that men with infertility were excluded from the study, we did not assess serum LH and FSH levels in our study population, nor did we measure their testicular volumes. As such we cannot exclude with certainty the presence of primary testiculopathies and therefore we cannot rule out preexisting hypogonadism that may be unrelated to metabolic age alteration. Moreover, the diagnosis of ED was based only on the IIEF-5 results and as such some patients with psychogenic ED may have been included. Finally, lifestyle factors that may interfere with sexual function such as cigarette smoking were not evaluated.

## Conclusion

MetAge is significantly inversely correlated with serum testosterone levels overall. In men < 40 years of age, higher MetAge seems to have a negative impact on sexual function. This association may serve as an additional motive towards adopting a healthy lifestyle among the general population. Further studies of larger sample size are required to confirm or dispute these results.

## Data availability statement

The raw data supporting the conclusions of this article will be made available by the authors, without undue reservation.

## Ethics statement

The studies involving human participants were reviewed and approved by Medical research center, Hamad Medical Corporation, protocol number 13453/13. The patients/participants provided their written informed consent to participate in this study.

## Author contributions

All authors contributed to the article and approved the submitted version.
